# Understanding how residents’ preferences for supervisory methods change throughout residency training: a mixed-methods study

**DOI:** 10.1186/s12909-015-0462-7

**Published:** 2015-10-16

**Authors:** Francisco Olmos-Vega, Diana Dolmans, Jeroen Donkers, Renée E. Stalmeijer

**Affiliations:** 1 Pontificia Universidad Javeriana, Carrera 7 # 40-62, Bogotá, Colombia; 2 Maastricht University, 6200 MD Maastricht, The Netherlands; 3 Anaesthesiology Department, San Ignacio Hospital, Carrera 7 N42-00 Fourth floor, Bogotá, DC Colombia

**Keywords:** Clinical Supervision, Postgraduate training, Cognitive Apprenticeship, Residency, Workplace learning

## Abstract

**Background:**

A major challenge for clinical supervisors is to encourage their residents to be independent without jeopardising patient safety. Residents’ preferences according to level of training on this regard have not been completely explored. This study has sought to investigate which teaching methods of the Cognitive Apprenticeship (CA) model junior, intermediate and senior residents preferred and why, and how these preferences differed between groups.

**Methods:**

We invited 301 residents of all residency programmes of Javeriana University, Bogotá, Colombia, to participate. Each resident was asked to complete a Maastricht Clinical Teaching Questionnaire (MCTQ), which, being based on the teaching methods of CA, asked residents to rate the importance to their learning of each teaching method and to indicate which of these they preferred the most and why.

**Results:**

A total of 215 residents (71 %) completed the questionnaire. All concurred that all CA teaching methods were important or very important to their learning, regardless of their level of training. However, the reasons for their preferences clearly differed between groups: junior and intermediate residents preferred teaching methods that were more supervisor-directed, such as modelling and coaching, whereas senior residents preferred teaching methods that were more resident-directed, such as exploration and articulation.

**Conclusions:**

The results indicate that clinical supervision (CS) should accommodate to residents’ varying degrees of development by attuning the configuration of CA teaching methods to each level of residency training. This configuration should initially vest more power in the supervisor, and gradually let the resident take charge, without ever discontinuing CS.

**Electronic supplementary material:**

The online version of this article (doi:10.1186/s12909-015-0462-7) contains supplementary material, which is available to authorized users.

## Background

The learning process of residents has traditionally been described as “a process of progressively *independent* delivery of patient care by a trainee, associated with a *decreasing level of supervision* by clinical supervisors” [1]. The medical education literature worldwide seems to embrace the view that clinical residency training should promote progressive independence, implying a corresponding phasing out of supervision, even though there is no empirical evidence regarding the effectiveness of this approach [1]. In fact, some of the literature on clinical supervision (CS) has sought to determine how this phasing out of CS should be effected and when completely independent resident practice should set in [2–5]. At the same time, the benefits of supervision have been lauded in numerous publications on medical education [6, 7] and various guidelines for effective supervision in both undergraduate and postgraduate settings have been published [8]. Curiously, these frameworks envisage CS for *all* students, irrespective of their level of training, and the idea of progressive independence is mostly absent [8]. It follows that little is known about how supervisors should adapt their teaching methods or behaviours to residents’ varying levels of experience and expertise.

The idea that supervision should be phased out is part of the traditional apprenticeship model in which novices are apprenticed to experts. Collins and colleagues rethought this model by introducing Cognitive Apprenticeship (CA) which rendered the processes involved in experts’ solving of complex cognitive tasks more explicit [9]. Through *modelling*, clinical supervisors show trainees how to perform a given task, emphasising the important elements that elicit a correct performance. In the next process, *coaching*, supervisors directly observe trainees performing the task and give effective feedback to improve their overall performance. These processes are complemented by *scaffolding*, during which trainees’ levels of expertise are assessed, and trainees are challenged with tasks that are tailored to these levels. In this, supervisors should know whether additional support is needed and, if so, when, but should also gradually fade this support as trainees become more skilled. In the process of *articulation*, clinical teachers induce trainees to provide the reasoning behind their decisions. While doing so, clinical teachers also promote *reflection*, a process that helps students understand their own strengths and weaknesses. Finally, *exploration* is the method in which trainees are encouraged to formulate learning goals and find ways to achieve these [9]. It is important to indicate that a clinical teacher may point out both strengths and weaknesses of a given trainee as part of specific feedback. However, by using articulation, the trainee learns to understand what the specific characteristics of his performance are that require improving and as such strengthening the learning experiences.

These processes, hereinafter referred to as CA teaching methods, can be divided into two groups: modelling, coaching and scaffolding on the one hand, and reflection, articulation and exploration on the other. Where the first relate to the traditional apprenticeship model and are supervisor-directed, the second can be coined resident-directed or self-directed [9].

Previous research has indicated that both clerkship students and clinical teachers greatly value the use of CA teaching methods during supervision in the clinical workplace [10, 11]. However, as mentioned before, no empirical evidence exists as yet as to how supervisors should adjust their behaviour to residents’ varying levels of expertise in the context of the CA model. Research on supervision in undergraduate education supports a developmental model of clinical teaching that is based on CA and in which the supervisory teaching methods move from modelling and creating a safe learning environment in the beginning, through coaching in a second phase, on to articulation and exploration in a third phase [11]. This theoretical model implies that undergraduate supervision is an ongoing process in which the teaching methods used by the supervisor change with the student’s level of expertise, from those that are mainly supervisor-directed to those that are mainly self-directed. Unlike the previously defined paradigm of postgraduate training [1], the undergraduate model does not suggest that clinical supervision should be discontinued in later stages of training. Instead, it proposes a variety of teaching methods that can be adjusted to the student’s needs and to the context throughout the training process, allowing the supervisor to provide continued supervision that warrants good patient care without being too dominant.

The CA model of undergraduate supervision has found resonance only in research on training in counselling and psychotherapy which supports a developmental model of supervision that accounts for trainees’ level of expertise [12]. This model progresses from intensive supervision and feedback for the beginner, to being collaborative and consultative for the advanced trainees, without discontinuing supervision, but changing its focus of action. An important feature is that it also incorporates trainees’ reflection into all levels of training [12].

The main hypothesis underpinning this study is that CS should be provided at all levels of residency training instead of phasing it out as independence at work progressively increases. What’s more, clinical supervisors should attune their teaching methods to residents’ level of training, gradually increasing their autonomy without depriving them of opportunities to extend their expertise. One advantage of residency programmes is that rotations span a significant period of time allowing students to be incessantly exposed to the same group of supervisors; this adds continuity to supervision and, theoretically, provides scope for students to be exposed to the whole string of teaching methods proposed by Collins [11]. The present study therefore seeks to investigate which teaching methods of the CA model junior, intermediate and senior residents prefer and why, and how these responses differ between groups.

The research questions are:

1. To which extent do residents prefer their supervisors to employ the different teaching methods of the CA model and does this differ according to years of residency?
2. What reasons do residents provide for their preferences?

## Methods

### Setting and participants

The study was conducted at San Ignacio Hospital, the main academic centre of Javeriana University in Bogotá, Colombia. The university has 19 residency programmes, with 301 students enrolled at the time the study was conducted (between December 2013 and February 2014). As literature suggests that learners can be more easily subjected to the whole string of CA teaching methods when they have the same supervisors for an extended period of time [10, 11], we only invited residents who had been enrolled in a residency programme for at least two months; this increased the likelihood of residents recognising the methods in the questionnaire. In Colombia, medical training consists of a 6-year undergraduate programme that ends with an internship in the final year. To be able to apply for a residency programme, all graduate students must consequently complete one year of rural community service. Residency programmes vary from 3 to 5 years depending on the specialty.

### Methodology

We used a mixed-methods design with concurrent collection of quantitative and qualitative data to answer the research questions [13]. The collection of quantitative data by means of a questionnaire allowed us to include a large sample of residents, whereas the qualitative data served to give us a better insight into the rationales behind the answers to the quantitative questions.

### Research team

The research team consisted of an anaesthesiologist pursuing a Master’s in Health Professions Education [14], two educationalists (DHJMD, RES) and one knowledge engineer (JD). As part of their mandatory research activities, three anaesthesiology residents assisted the first author in collecting the questionnaires.

### Instrument

We used a validated Spanish version of the Maastricht Clinical Teaching Questionnaire (MCTQ) [15] to measure residents’ preferences with regard to the teaching methods of the cognitive apprenticeship model. The MCTQ was developed by Stalmeijer et al. based on the CA model as described by Collins, and has been validated as a tool for the evaluation of clinical teaching quality and as a source of feedback regarding clinical supervisors’ performance [16, 17]. The questionnaire has 15 items that are rated using a 5-point Likert scale, including an overall rating of the clinical teaching quality. The items are grouped according to Collins’ model into the factors *modelling*, *coaching*, *articulation*, *exploration* with the addition of an element about the creation of a *safe learning environment* as this has been associated with successful learning in clinical environments [8] (see Additional file 1).

### Process

The first author contacted both the residents’ current superior and the coordinator of each programme in order to schedule a half-hour session to complete the questionnaires. These meetings took place in the absence of supervisors. At the beginning of the session, we explained the purpose of the study and gave residents the opportunity to ask questions. Subsequently, we asked each resident to fill in a MCTQ, rating the importance of each item in the context of his or her current year of residency. We used a 5-point Likert scale on which the numerical values ranged from 1 being ‘least important to my level of training’ to 5 ‘being most important to my level of training’. The last question of the questionnaire asked residents to describe “Which of the previous factors-modelling, coaching, articulation, exploration and safe learning environment-do you deem the most important to your learning process in view of your current year of residency and why?”.

### Ethical considerations

We obtained ethical approval from the ethics research council of San Ignacio Hospital and Javeriana University before the beginning of the study. Informed consent was obtained from all the participants. Identifying information provided on each questionnaire was coded so as to guarantee residents anonymity of the results.

### Statistical analysis

We conducted a stepwise analysis of each factor as our aim was to compare the CA teaching methods. To this end, we computed means of all items pertinent to each factor. Before analysing the data, responses were grouped into three categories according to their origin: juniors (year 1 residents), intermediates (year 2 residents) and seniors (year 3–5 residents; our sample included only 1 year 5 resident). The reason for clustering residents this way was our interest in analysing preferences chronologically based on residency year. However, given the lack of Year 4 residents we decided to group all third and fourth year residents to avoid too much discrepancy in terms of size of the groups. We obtained descriptive statistics for all computed variables.

We ran separate one-way ANOVA tests for each cognitive apprenticeship teaching method of the MCTQ comparing the means for each level of training. We also ran two planned contrasts, the first one being the difference between junior residents on the one hand and the combination of intermediate and senior residents on the other, and the second one being the difference between intermediate and senior residents.

To allow for a comparison of the teaching method residents deemed most important across levels of training, we obtained crosstabs for these two variables. As the count for some cells of the crosstabs was less than 5, we calculated likelihood ratios for categorical variables, and standardised scores to determine trends of the main factors.

All analyses were performed using SPSS version 19 for MAC OS. P values of less than 0.05 were considered significant.

### Qualitative analysis

In a single document we ordered the answers to the open-ended question, first according to level of training and subsequently according to teaching method of the CA model. We performed thematic analysis as per the stepwise approach suggested by JW Creswell [18]. We extracted codes from the data and then grouped them into themes.

## Results

Among the residents who agreed to participate, all departments and all levels of training were represented (see Table 1). A total of 211 in 301 residents completed the questionnaires (response rate: 71.4 %), whereas 45.1 % of the respondents were male, and 54.9 % were female. 206 residents answered which was the most preferred teaching method, however only 65.1 % junior, 57.5 % intermediate and 25 % senior residents that completed the MCTQ answer the open-ended question fully.

**Table 1:** Contingency table of residency programme and level of training

	Level of training			
Residency Programme	Junior	Intermediate	Senior	Total
Anaesthesiology	6	6	6	18
General Surgery	6	2	8	16
Plastic Surgery	1	1	1	3
Gynaecology and Obstetrics	4	2	2	8
Genetics	2	2	1	5
Geriatrics	3	5	3	11
Family Medicine	4	5	5	14
Internal Medicine	11	11	11	33
Emergency Medicine	4	6	3	13
Neurosurgery	0	1	4	5
Neurology	1	2	2	5
Ophthalmology	2	1	0	3
Otorhinolaryngology	2	2	2	6
Orthopaedics	7	3	10	20
Pathology	2	3	2	7
Paediatrics	0	5	7	12
Psychiatry	6	6	5	17
Radiology	5	3	6	14
Urology	0	0	1	1
Total	66	66	79	211
Mean age	27	28	28	

### Residents’ preferences as to the type of CA teaching method used

All CA teaching methods of the MCTQ were rated highly by residents at all levels of training (see Table 2). From an analysis of the Likert-scale questions about the preferred teaching method, modelling emerged as the only teaching method that received ratings that differed significantly across the three levels *F* (2, 211) = 7.02, *p* = .001, although the effect size was small, ω = 0.2. Further analysis unveiled a significant linear trend, *F* (1, 211) = 8.47, *p* = .004, of residents attaching less and less weight to modelling as they progress through their residency programme. Planned contrast confirmed that junior residents indeed preferred modelling more in comparison to their senior colleagues *t* (211) = −3.70, *p* = .000, *r* = 0.24. Intermediate residents, by extension, did not differ significantly from senior residents in the value they attached to modelling, *t* (211) = −0.24, *p* = .809, *r* = 0.01.

**Table 2:** Mean residents’ preferences according to level of training for each Maastricht Clinical Teaching Questionnaire (MCTQ) factor

MCTQ Factor	Level of training		
	Junior	Intermediate	Senior
Modelling	4.6 (0.44)	4.39 (0.77)	4.2 (0.75)
Coaching	4.57 (0.47)	4.58 (0.48)	4.43 (0.54)
Articulation	4.28 (0.82)	4.12 (0.94)	4.2 (0.85)
Exploration	4.02 (1.06)	3.91 (1.17)	4.12 (1.02)
SLE	4.89 (0.28)	4.76 (0.36)	4.74 (0.60)

Note: *SLE* = Safe Learning Environment, Results are presented in means, standard deviations are in brackets

**Fig. 1:**
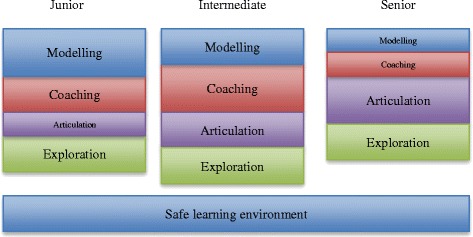
Developmental model of clinical supervision according to resident’s preferences. The bigger the box, the more preferred the factor is

When asked which CA teaching method they deemed most important in regard to their level of training, replies appeared to be significantly contingent on the level of training (see Table 3 and Fig. 1). The standardised residuals of each cell revealed that among junior residents modelling was most preferred, and articulation least; intermediate residents had a strongest preference for coaching, while there was no particular teaching method they preferred the least; senior residents, in contrast, preferred articulation most, and modelling and coaching least. The differences were significant, Λ (8) = 59.86, *p* < .001.

**Table 3:** Contingency table of level of training and the most preferred MCTQ teaching method (TM)

Level of Training		Teaching Method					
		M	C	A	E	SLE	Total
Junior	Count	24	29	4	2	7	66
	% within TM	77.4	31.5	11.8	14.3	20	32 %
	Std Residual	**4.5**	−0.1	**−2.1**	−1.2	−1.3	
Intermediate	Count	4	40	7	3	8	62
	% within TM	12.9	43.5	20.6	21.4	22.9	30.1 %
	Std Residual	−1.7	**2.3**	−1.0	−0.6	−0.8	
Senior	Count	3	23	23	9	20	78
	% within TM	9.7	25	67.6	64.3	57.1	37.9 %
	Std Residual	**−2.6**	**−2.0**	**2.8**	1.6	1.9	
Total		31	92	34	14	35	206

*Note*: Standardised residuals (Std Residual) in bold correspond to *p* < 0.05. Positive values of these standardised residuals correspond to the teaching methods that are more preferred and negative values to those that are the less preferred ones

*M* = Modelling, *C* = Coaching, *A* = Articulation, *E* = Exploration, *SLE* = Safe Learning Environment

**Table 4:** Residents’ Preferences with regard to the cognitive apprenticeship teaching methods. Main themes according to level of training

Junior	Intermediate	Senior
Supports skills acquisition by using Modelling	Support growing independent practice by using Coaching	Fix gaps in competence development by using exploration
“…*because at this point rather than learning the theory stuff what I want is to acquire clinical abilities and skills when approaching the patients*” (Junior Resident #26).	“*I think this is the most important one*, *because it allows me to perform the clinical activities independently whilst receiving supervision and feedback in order to improve the abilities step by step*.” (Intermediate Resident #3)	“*In this moment my training is almost complete and I think about what am I lacking to face a competitive working market*” (Senior Resident # 75).
Overcoming deficiencies by using Coaching	Help expand knowledge base and engage in dialogue with supervisor by using Articulation	Help expand knowledge base and engage in dialogue with supervisor by using articulation
“*I think that in this stage of training it would help me to have more feedback with regard to the quality*, *pertinence and rationally of my actions*” (Resident (Junior) #34)	“*Laying the foundations of my actions and exploring my strengths and weaknesses allow me to develop and improve my clinical criterion and my decision*-*making skills*” (Resident (Intermediate) #22)	“*In this year I think that one already has enough knowledge and training to show what do you know and what you don*’*t*.” (Senior Resident #24)
“*At this level of academic training*, *initial phase*, *I will be better helped by permanent feedback in order to fix the fails and to strengthen the right choices*…” ( Junior Resident #71)	“*Because I consider that it motivates you to make your own decisions and it makes you feel important in the patient care*” (Intermediate Resident #65)	“*It allow us not only to answer questions but also to formulate them*” (Senior Resident #54)
Encourage participation by creating a Safe Learning Environment		Gain confidence in own performance by not be coerced using a Safe learning environment
“ *I think that the supervisor*-*trainee relationship is very important. If the supervisor creates an environment of trust and respect*, *it is possible to loose a little bit that formality and rigidity a teacher has and one can ask questions*, *formulate doubts and even have more security as a student*..” (Junior Resident #80)		“…*this is the moment to give confidence and respect* (*to the resident*) *in terms of what has been taught and modeled*, *this can only be reflected in a safe learning environment that not coerce our free performance*.” (Senior Resident #18)

### Exploring reasons behind these preferences

To gain insight into residents’ motives for preferring certain teaching methods to others, we analysed the answers to the open-ended question asked at the end of the MCTQs. The analysis below presents the main themes that emerged for each group of residents (See Table 4).

### Junior residents

The most important concern for junior residents was to have good foundations in terms of knowledge and clinical skills. They considered it very important that these foundations were acquired rapidly such that they could avail themselves fully of the learning opportunities the workplace offered. Modelling and coaching were perceived as the tools that could effectively address these concerns: modelling helped residents, under the wing of their clinical supervisors, to construct solid knowledge and skills foundations that support the acquisition of clinical expertise, whereas coaching encouraged them to become better aware of their strengths and weaknesses and to overcome initial deficiencies. An important aspect of these teaching methods, in the view of junior residents, was that, being under constant supervision, they did not yet have to work completely independently, which minimised errors in patient care. A precondition, however, was that learning took place in a safe environment that fostered their active participation in the patient-care process. In summary, modelling and coaching were considered crucial for a rapid construction of solid clinical skills, for encouraging residents’ reflection about their strengths and weaknesses and for minimising errors that would arise from unsupervised practice.

### Intermediate residents

To intermediate residents, on the other hand, it was very important that they could build upon their previously acquired knowledge and skills and grow in their role. In their view, increased independence would help them achieve this. At the same time, however, most of the intermediate residents also appreciated receiving feedback on their independent performance. They therefore chose coaching as the most important teaching method as it allowed them to work independently and receive feedback as well. This feedback was needed to improve their skills and knowledge step by step. Intermediate residents further regarded coaching as a bridge between modelling and articulation: together with modelling, it provided the right basis for the resident to be able to occupy a more central role in patient care later on by means of articulation. Articulation, moreover, was perceived as a method that helped them develop decision-making skills and expand their knowledge base. Yet, what figured as most important at this stage of training was a combination of coaching and independent practice.

### Senior Residents

For residents in the final years of training, the most important concern was to consolidate knowledge and skills in order to be prepared for future practice. In this discourse, exploration arose as a method of crucial importance, for it helped senior residents to fix gaps in their competence development. A safe learning environment, which, moreover, in the view of senior residents should not be authoritative, was considered essential in pursuing this goal. Articulation was perceived as the teaching method that would nurture such a safe learning environment. In general, senior residents set great store by articulation which allowed them to expand their knowledge base and engage themselves in dialogue with the supervisor. As a result, they could participate more actively in the patient-care process while still being under supervision that was not authoritative.

## Discussion

Previous studies have explored residents’ perceptions of CS. While some of these focused on the intensity of supervision and residents’ overall satisfaction with the supervision [19], others have zoomed in on supervisor characteristics that residents preferred most [20–22]. Yet, none of these has sought to identify how preferences with regard to the teaching methods used in CS differed according to level of training, nor have they used a clear theoretical framework to explore these perceptions.

In this study we have sought to determine residents’ preferences with regard to the teaching methods used in CS, and to compare these across levels of training. To this end, we used the teaching methods of the CA model as the main theoretical framework [9]. Our results indicate that CS should accommodate to residents’ varying degrees of development by attuning the configuration of CA teaching methods to each level of residency training. This configuration should initially vest more power in the supervisor (by using methods such as modelling and coaching) and gradually let the resident take charge (by using methods such as exploration and articulation), without ever discontinuing CS (see Fig. 1). Qualitative data confirmed and elucidated our quantitative findings, which revealed how this transition should be effected. The analysis also yielded valuable information on how to use each teaching method at each level of training so that the concerns of residents would be effectively addressed. The recommended approach varied from helping residents to construct solid knowledge and skills foundations, through to having them perform the task independently and providing effective feedback afterwards, to finally end with having them actively participate in patient care by engaging them in meaningful dialogue with the supervisor. In this last stage it is important to ensure that learning take place in an environment that is not intimidating the resident.

Our results are consistent with the CA teaching model for undergraduate students reported earlier [16] and could extrapolate its application to postgraduate settings. They also reverberate the developmental model of supervision for counselling and psychotherapy students [12] in terms of how supervisory teaching methods should change with students’ level of training. By exploring residents’ preferences, we also put to the test the traditional paradigm that favours a gradual fading of CS in the course of residency training [1, 3]. Our findings indicate that by customising constellations of CA teaching methods, specific needs of residents can be targeted at each level of training. What’s more, such constellations would allow supervisors to provide on-going supervision that warrants patient safety, and at the same time encourage residents to actively participate in patient care while retaining their autonomy.

In this discourse, however, some limitations are worthy of mention. First, although our hypothesis was supported by both statistical and thematic analyses, we only gauged residents’ preferences with regard to supervisor behaviour, not the actual effectiveness of CS. Admittedly, our study provides a starting point for how to structure the CS teaching methods according to residents’ level of expertise. However, these results should be complemented by studies that measure the impact of this developmental model on residents’ learning and development of expertise, especially in the long run. Second, future research should also explore more in-depth the perceptions of both residents and supervisors with regard to the value of each CA teaching method in relation to the various levels of training. Our study indeed revealed a high level of appreciation of all methods across all levels, but did not go into all aspects exhaustively: certain factors, such as exploration for instance, still remain obscured. An in-depth understanding of changes in appreciation according to residents’ level of training would yield further insights with regard to how the cognitive apprenticeship model can guide CS during residency training. Third, our methodology did not allow us to determine the influence of specific factors on residents’ preferences, such as individual characteristics, complexity of the task or even the specific workplace context in which this task is developed. Future research should explore these influences in-depth, in order to make a better informed decisions with regard to how to make the transition from one teaching method to the other.

The main practical implication arising from our results is that teaching methods of the CA model could be used at all levels of training, based on residents’ preferences. Furthermore, we propose that CS should accommodate to residents’ varying degrees of development by attuning the configuration of CA teaching methods to each level of residency training. This configuration should initially vest more power in the supervisor and gradually let the resident take charge by using self-directed teaching methods, without ever discontinuing CS. Consequently, our findings could be used to inform the training of clinical supervisors in faculty development programmes and curriculum design in postgraduate education. It could also be the first step to strike a balance between providing CS while simultaneously increasing residents’ independence.

## Conclusion

This survey into residents’ preferences with regard to the use of teaching methods during CS expands the theories of workplace learning and teaching during residency [23, 24]; moreover, it yields important insights about how the CA model can be wielded to guide supervisor behaviour [8] and improves our understanding of how such practices should be adjusted to residents’ varying levels of expertise [1]. What’s more, it represents the first step in taking up the challenge of providing continuous supervision while encouraging resident autonomy.

## Additional file

Additional file 1: **Maastricht Clinical Teaching Questionnaire.** (DOC 48 kb)
