# Supplementary material for: Understanding how residents’ preferences for supervisory methods change throughout residency training: a mixed-methods study
Source: BMC Med Educ. 2015 Oct 16;15:177. doi: 10.1186/s12909-015-0462-7 (PMC4609127; doi:10.1186/s12909-015-0462-7)
Supplement: Additional file 1: — Maastricht Clinical Teaching Questionnaire. (DOC 48 kb) [file 12909_2015_462_MOESM1_ESM.doc]

# Additional file 1.

**Maastricht Clinical Teaching Questionnaire**

**Residents’ preferences with regard to Cognitive Apprenticeship teaching methods.**

**Name: Age: Sex:**

**Residency programme:**

**Current year of residency:**

Please rate the importance of each of the next items IN RELATION TO YOUR CURRENT LEVEL OF RESIDENCY: 1 if you consider it less important, and 5 if you deem it more important. Please give an overall rating of your preference and do not focus on specific or complex scenarios. Read each item carefully and be honest about your answer.

|  |  |  |  |  |  |
| --- | --- | --- | --- | --- | --- |
| **Modelling.** |  |  |  |  |  |
| Consistently demonstrated how to perform clinical skills. | 1 | 2 | 3 | 4 | 5 |
| Created sufficient opportunities for me to observe him/her. | 1 | 2 | 3 | 4 | 5 |
| Served as a role model as to the kind of doctor I would like to become. | 1 | 2 | 3 | 4 | 5 |
| **Coaching.** |  |  |  |  |  |
| Gave useful feedback during or immediately after direct observation of my patient encounters. | 1 | 2 | 3 | 4 | 5 |
| Adjusted his/her teaching activities to my level of experience. | 1 | 2 | 3 | 4 | 5 |
| Offered me sufficient opportunities to perform activities independently. | 1 | 2 | 3 | 4 | 5 |
| **Articulation**. |  |  |  |  |  |
| Asked me to provide a rationale for my actions. | 1 | 2 | 3 | 4 | 5 |
| Asked me question aimed at increasing my understanding. | 1 | 2 | 3 | 4 | 5 |
| Stimulated me to explore my strengths and weaknesses. | 1 | 2 | 3 | 4 | 5 |
| **Exploration.** |  |  |  |  |  |
| Encouraged me to formulate learning goals. | 1 | 2 | 3 | 4 | 5 |
| Encouraged me to pursue my learning goals. | 1 | 2 | 3 | 4 | 5 |
| **Safe Learning environment.** |  |  |  |  |  |
| Created a safe learning environment. | 1 | 2 | 3 | 4 | 5 |
| Was genuinely interested in me as a student. | 1 | 2 | 3 | 4 | 5 |
| Showed that he/she respected me. | 1 | 2 | 3 | 4 | 5 |

Which of the previous factors -modelling, coaching, articulation, exploration and safe learning environment- do you deem the most important to your learning process in view of your current year of residency and why?
